# P-888. Variability in Durations of Therapy for Gram-Negative Bloodstream Infections Across U.S. Hospitals

**DOI:** 10.1093/ofid/ofae631.1079

**Published:** 2025-01-29

**Authors:** Jesse Sutton, Jae Hyoung Lee, Drew Engers, Karen Fong, Alok Gupta, Sara M Karaba, Anurag Malani, Christopher McCoy, Kelly E Pillinger, Katelyn Quartuccio, Judianne C Slish, Pranita Tamma, Timothy C Jenkins

**Affiliations:** George E Whalen Veterans Affairs Medical Center & University of Utah, Salt Lake City, Utah; Johns Hopkins, Baltimore, Maryland; Trinity Health, Ann Arbor, Michigan; University of Utah Health, Salt Lake City, Utah; University of Rochester Medical Center, Rochester, New York; Johns Hopkins University, Baltimore, MD; Trinity Health Michigan, Ann Arbor, Michigan; Beth Israel Deaconess Medical Center, Boston, Massachusetts; PRIME Education, Pittsford, New York; University of Rochester Medical Center, Highland Hospital, Rochester, NY; University of Rochester Medical Center, Rochester, New York; Johns Hopkins School of Medicine, Baltimore, MD; Denver Health, Denver, Colorado

## Abstract

**Background:**

There is a paucity of data on durations of antibiotic therapy utilized in clinical practice for patients with Gram-negative bloodstream infections (GN-BSI). The objectives of this study were to describe treatment durations for patients with GN-BSI and evaluate variability across U.S. hospitals.

Histogram of antibiotic treatment durations

**Figure:**
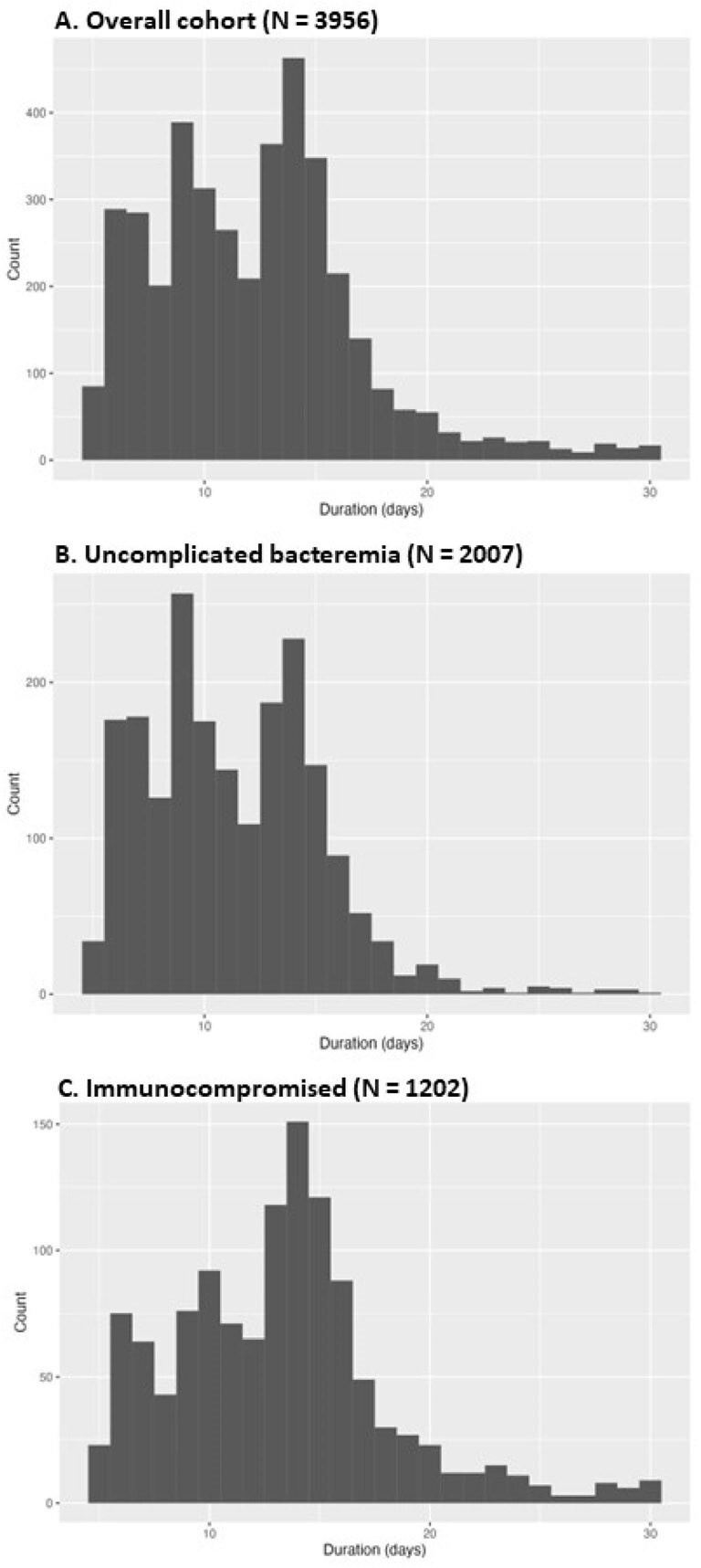

**Methods:**

This was a retrospective cohort study of patients hospitalized at 24 hospitals (16 academic, 4 community, and 4 Veterans Affairs) with a GN-BSI between January 1, 2019 and December 31, 2019. Episodes of GN-BSI from a urinary, intra-abdominal, hepatobiliary, vascular catheter, skin and soft tissue, or respiratory source were included while those associated with a bone, joint, central nervous system, or endovascular infection or prostatitis were excluded. Subgroups of interest were cases of uncomplicated bacteremia and those with underlying immunosuppression. Uncomplicated bacteremia was defined as: (1) achievement of source control, when applicable; (2) absence of immunosuppression; and (3) resolution of fever and hypotension within 3 days.

Variability in median duration of therapy across hospitals

**Figure:**
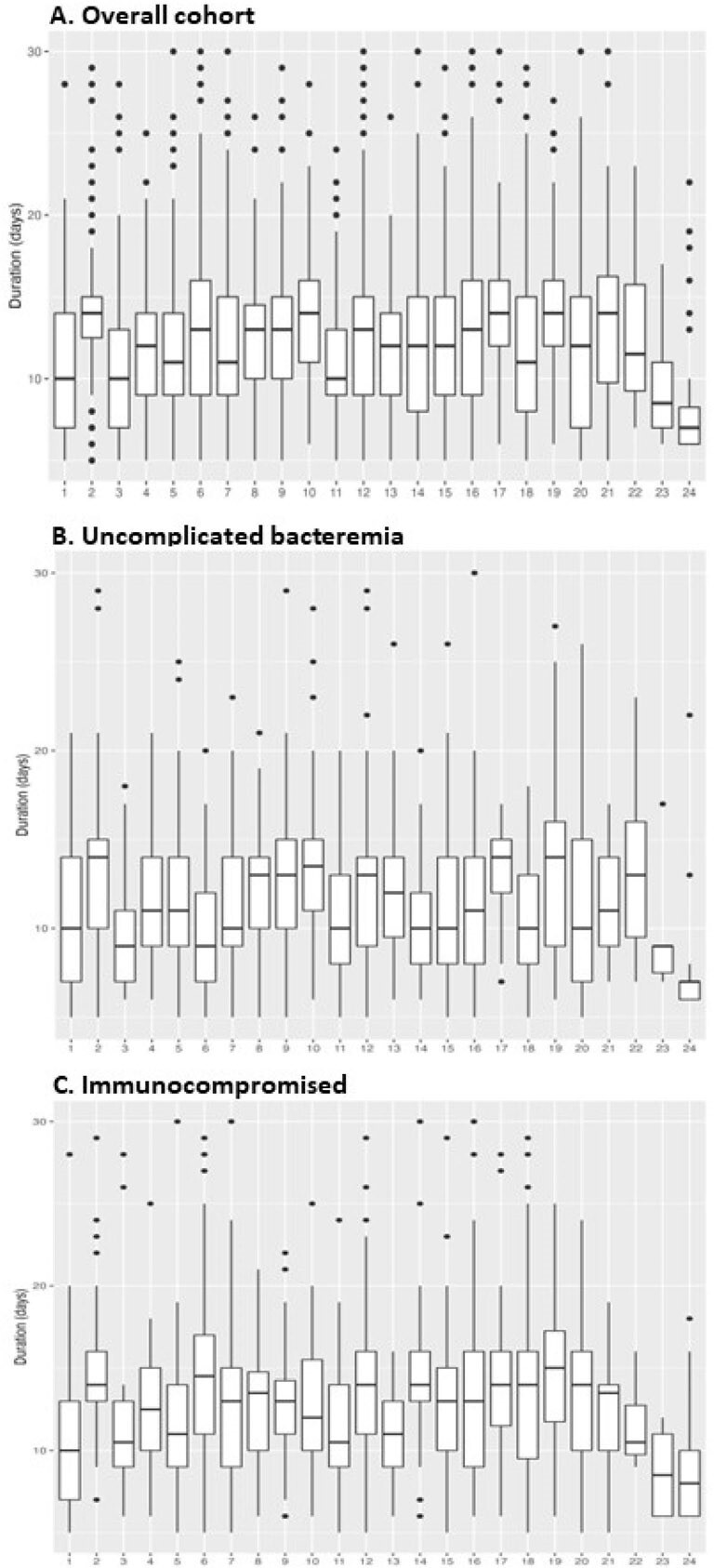

**Results:**

3,956 episodes of GN-BSI were included, of which 2,007 were uncomplicated bacteremia and 1,202 involved underlying immunosuppression. Urinary tract (55%), intra-abdominal (14%), and hepatobiliary (12%) sources accounted for the majority of the infections, while *E. coli* (53%), *K. pneumoniae* (12%), and *P. aeruginosa* (8%) were the most common pathogens. The median duration of therapy in the overall cohort and uncomplicated and immunocompromised subgroups was 12 (interquartile range [IQR] 9 - 15) days, 11 (IQR 8 - 14) days, and 11 (9 - 14) days, respectively (**Figure 1**). In the overall cohort, there was marked variation in treatment durations across hospitals, with the median duration as low as 5 (IQR 3 - 7) days and as high as 14 (IQR 10 - 17) days (**Figure 2**). Similar variability was observed in the uncomplicated bacteremia and immunocompromised subgroups.

**Conclusion:**

There is marked variation in treatment durations for GN-BSI across U.S. hospitals. Dissemination of interventions to promote evidence-based short durations of therapy for uncomplicated bacteremia and elucidating the optimal duration of therapy in immunocompromised patients will be necessary to standardize care and reduce unnecessary antibiotic use.

**Disclosures:**

**Sara M. Karaba, MD, PhD, MHS**, Entasis: Advisor/Consultant **Kelly E. Pillinger, PharmD**, AHFS: Contractor **Judianne C. Slish, Pharm.D., BCPS**, Glaxosmithkline Pharmaceuticals: Honoraria

